# An Effective Primary Treatment Using Radiotherapy in Patients with Eyelid Merkel Cell Carcinoma

**DOI:** 10.3390/curroncol30070468

**Published:** 2023-07-02

**Authors:** Marie Boileau, Manon Dubois, Henry Abi Rached, Alexandre Escande, Xavier Mirabel, Laurent Mortier

**Affiliations:** 1 CHU Lille, Department of Dermatology, University of Lille, F-59000 Lille, Francehenry.abirached@chu-lille.fr (H.A.R.);; 2 Department of Medicine, University of Lille, H.Warembourg, F-59000 Lille, France; 3 CRIStAL Laboratory, UMR 9189, University of Lille, F-59655 Villeneuve d’Ascq, France; 4 CHU Lille, University of Lille, Inserm, U1189—ONCO-THAI—Assisted Laser Therapy and Immunotherapy for Oncology, F-59000 Lille, France; 5 Oscar Lambret Comprehensive Cancer Center, Universitary Department of Radiation Oncology, F-59000 Lille, France; x-mirabel@o-lambret.fr

**Keywords:** Merkel cell carcinoma, curative radiation, eyelid, skin cancer, margin, eyelid, irradiation

## Abstract

Background: Merkel cell carcinoma (MCC) is a rare type of neuroendocrine tumor. Palpebral localization represents 2.5% of MCCs. Surgery is not always possible due to the localization or comorbidities of elderly patients. We hypothesized that radiotherapy (RT) alone could be a curative treatment in patients contraindicated for oncological surgery. Methods: We performed a retrospective monocentric study of patients with localized eyelid MCC treated with curative intent using curative radiotherapy. Results: Overall, 11 patients with histologically confirmed eyelid MCC were treated with curative radiotherapy. The median age was 77 years old (range: 53–94). Curative RT was decided mainly due to difficult localization and significant co-morbidities. The median lesion dose was 57 Gy (range: 47–70). Most patients had adjuvant lymph nodes irradiation with a median dose of 50 Gy (*n* = 9; 82%). The median follow-up was 62 months (6–152 months). None of the seven deaths were MCC-related. None of our patients relapsed during follow-up. Side effects related to radiotherapy were mild (no grade ≥ 2) and rare (*n* = 3, 21%). Conclusion: Our data suggest that curative radiotherapy is an effective and safe treatment for Merkel cell carcinoma of the eyelid and periocular region. Radiotherapy alone allows limiting the aesthetic and functional sequelae in elderly and comorbid patients who are contraindicated for oncological surgery.

## 1. Introduction

Merkel cell carcinoma (MCC) is a rare but aggressive neuroectodermal primary skin cancer. Its incidence ranges from 0.11 to 0.4 cases per 100,000 in Europe. This aggressive tumor has a high risk of recurrence at around 25 to 50%; overall survival at 5 years ranges from 41 to 70% [1,2,3,4,5]. Although cellular etiology of MCC is unclear [6], risk factors include being elderly [7], UV exposure and immunodepression [8,9,10]. Its incidence is increasing over time due to an aging population, higher amounts of sun exposure, and better diagnostic methods and case registrations [11]. MCC is associated with chronic lymphocytic leukemia (CLL), small lymphocytic lymphoma (SLL), non-Hodgkin lymphoma (NHL), skin squamous cell carcinoma, and other cancers [7,12,13]. MCC presents as an asymptomatic rapidly growing, erythematous, or purple nodule in sun-exposed skin, mainly on the head or neck.

Nearly 200 cases of MCC located on the eyelid have been reported [14], which represent 2.5% of all MCCs [15]. The clinical characteristics of primary eyelid MCC are no different from other locations. Unlike basal cell carcinoma, MCC occurs twice as frequently on the upper eyelid [14,16]. Macroscopically, MCC can be misdiagnosed as a dermal cyst, chalazion, basal cell carcinoma, nodular angiosarcoma, or metastasis [16]. A size of 20 mm is usually used to determine the classification of MCC. Most of the previously reported orbital and eyelid MCCs had a diameter of 20 mm or less [17], with a median size of 15 mm [14]. This led to the use of the eighth edition of the American Joint Committee on Cancer classification criteria for eyelid carcinoma rather than the MCC criteria to achieve a more consistent T-category designation [18,19,20].

The diagnosis requires histological validation, using immunohistochemistry including CK-20 and TTF-1 immunomarkers to avoid misdiagnosis with lymphoma or small-cell lung carcinoma metastasis [21,22,23]. Eyelid MCC has a 20% risk of nodal metastasis on initial diagnosis [19]. Sentinel lymph node biopsy (SLNB) is recommended in Europe [23,24], but due to the age of patients and poor general estate, SLNB is rarely carried out on these patients [14]. Because of early and rapid discomfort, eyelid lesions are diagnosed earlier and have a better prognosis [14].

The standard care of MCC is wide local excision of the lesion with a negative lateral margin of 20 mm. An adjuvant radiation therapy to the primary tumor site is also recommended [21,23]. Without a clearly identified recommendation, a complete excision with a margin of at least 5 mm seems acceptable on the eyelid. [18,25] Mohs’ micrographic surgery may also be an appropriate method [23,26]. If the primary tumor is small, i.e., <10 mm, and widely excised with no other adverse risk factors such as lymphovascular invasion or immunosuppression, observation of the primary site may be considered. RT of the nodes is necessary when there is a risk of a false-negative nodal evaluation. RT should also be discussed in cases of profound immunosuppression [21,23,24].

Wide local excision may have aesthetic and functional impacts on patients. Furthermore, surgery has a high complication risk in elderly patients with many comorbidities. Indeed, our team previously reported that curative external beam radiotherapy (EBRT) yields acceptable oncological results for early MCC. Both studies of Mortier et al. and Pape et al. found no statistical differences in overall and disease-free survival between surgery plus radiotherapy or EBRT [27,28]. More recently, Dubois et al. compared 31 patients treated with surgery with a 2 cm clear margin followed by adjuvant radiotherapy (RT) and 53 patients treated with EBRT. No statistical difference was found for nodal relapse, metastatic relapse, disease-free survival, or overall survival. Hence, EBRT for early MCC leads to a similar oncological outcome as combined treatment, with fewer aesthetic sequelae [29].

We conducted a retrospective study to describe the management and the outcome of patients with eyelid Merkel cell carcinomas treated by curative radiotherapy.

## 2. Materials and Methods

### 2.1. Population

We reviewed the medical records of patients with eyelid MCC treated in our university center of Lille from 1999 to 2019. Diagnosis was confirmed histologically in all cases with a skin biopsy sample revealing a positive immunohistochemical staining for CK7-negative patients, and CK20- and neuron-specific enolase-positive patients. We included patients with localized disease, presenting as a unique tumor. Eyelid localization included the eyelid and the eyebrow. We excluded patients with localized disease in other places different from this location. Patients were included regardless of comorbidities, including hematological diseases. Lymph node involvement was assessed via imaging (either by ultrasound or CT scan). We excluded patients treated with surgery with a ≥0.5 cm margin or patients with lymph node metastasis. We staged the disease using the AJCC classification of Merkel cell carcinoma and eyelid carcinomas.

### 2.2. Treatment

MCC primary tumors were surgically excised to achieve negative surgical margins when possible. No chemotherapy or immunotherapy were performed. All patients received curative radiotherapy. The radiotherapy technique was variable according to treatment centers, and according to available radiation techniques at the time of treatment for all patients (3D, intensity-modulated radiotherapy, electron radiotherapy).

### 2.3. Statistical Analysis

Data are expressed as numbers (percentages) for categorical variables, and median for quantitative variables.

### 2.4. Ethics

There was no opposition from any of the patients included in the study for the use of their anonymized medical data, according to Jarde’s Law (March 2012) for retrospective data publications. This study has been declared and accepted by the CNIL (Commission Nationale Informatique et Libertés), the organization in charge of the ethical use of data collected for scientific purposes in France (DEC20-135)

## 3. Results

### 3.1. Patients’ Characteristics

A total of 11 patients with eyelid MCC were included. Most of them were women (7/11, 64%) with a median age of 77 years old [range 53–94 yo]. One patient had a history of Hodgkin lymphoma. The median size of the tumor was 13.2 mm [5–20 mm]. Most of the lesions were at stage T2N0M0 (6/11, 55%). Localizations were the eyelid for six patients (55%), more particularly, the upper eyelid (5/6, 83%), and on the eyebrow for the last five patients (5/11 (45%)) (Table 1).

### 3.2. Treatments

All patients were treated via curative EBRT. Three patients had a surgical biopsy with excision with insufficient margins of 0.1 cm. Of the six patients with an eyelid localization, five received curative radiotherapy (86%), and only one underwent surgical biopsy with excision with insufficient margins of 0.1 cm. None of our patients needed exenteration regardless of the treatment strategy or the localization of the eyelid. None had sentinel lymph node biopsy. None of our patients were treated with Mohs micrographic surgery (Table 2).

### 3.3. Irradiation

The median dose of radiation on the lesion was 57 Gy (47–70 Gy). For the six patients with MCC of the eyelid, the median dose was 57 Gy (47–70 Gy). Four of these six patients treated with radiotherapy without surgery were given a boost. The median dose of the boost was 12 Gy (8–20 Gy). Most of the patients had lymph node irradiation (9/11; 82%). The median dose delivered to the lymph nodes was 50 Gy (43–51). The techniques used were electrons, orthovoltage radiotherapy, 3D radiotherapy,y and IMRT. Five patients were treated with hypofractionned radiotherapy (3/11; 27%), with the median number of fractions being 15 [10,11,12,13,14,15,16,17,18].

### 3.4. Outcomes

**Figure 1:**
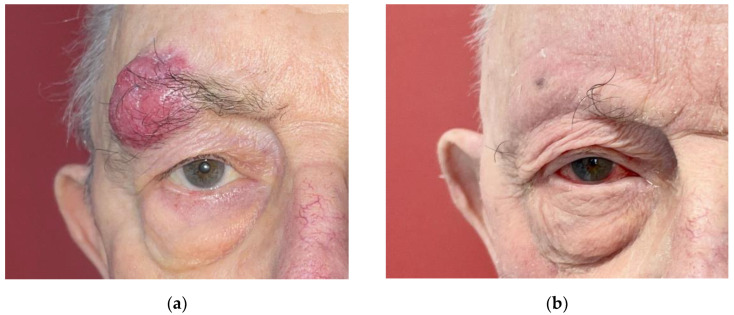
A 78-year-old patient with lesion of the right eyebrow (**a**) before treatment and (**b**) 10 days after curative irradiation: transient conjunctivitis grade 2 and complete resolution of the lesion.

**Figure 2:**
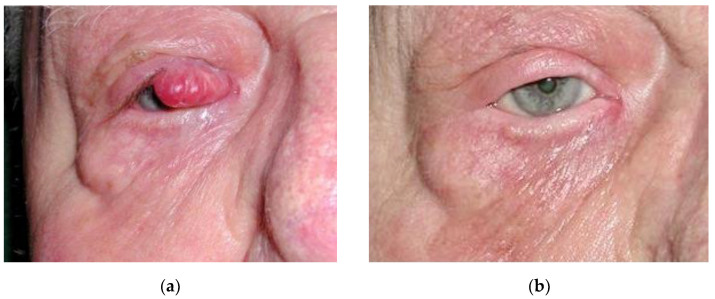
A 75-year-old patient 4 years after a treatment of 20 mm Merkel cell carcinoma of the right eyelid by curative radiotherapy. (**a**) Before treatment; (**b**) after treatment. Side effects were mild, only manifesting as alopecia of eyelashes and discreet hypochromia of the eyelid skin.

The median follow-up was 62 months (6–152). No patient had a local or metastatic relapse. A total of seven patients (64%) died during the follow-up; all were free of disease at the time of death. Causes of death were cardiac, infectious, or related to advanced age. Side effects of radiotherapy were mild and rare (3/11; 27%). The reported side effects were: alopecia of the eyebrow, radioepithelis, conjontivitis, and epistaxis (Table 2, Figure 1 and Figure 2).

## 4. Discussion

The management of eyelid Merkel cell carcinoma is mainly based on cases series [14,16,18,25]. Around 200 cases have been published and recently reviewed [14]. Data from randomized trials are not available due to the rarity of MCC. When the primary tumor is located on the eyelid, broad excision with a 20 mm margin is rarely feasible. Excision with a 5 mm margin to achieve tumor-free margin is accepted. The use of adjunctive radiotherapy is up to practitioners’ appreciation, although it is highly recommended by some authors [14,18,25].

However, some elderly patients may not be eligible for such surgery because of comorbidities and/or localization or extension of the tumor. The question then arises: what alternative treatment can be proposed to these patients who are at risk? In the present study, we report the results of 11 patients (77 years old) with localized eyelid MCC who were contraindicated for oncological surgery and treated with curative radiotherapy (RT). Our data suggest that curative RT is an effective and safe option for the treatment of eyelid MCC in elderly and comorbid patients. Curative radiotherapy provides good oncological outcomes and allows limiting aesthetic and functional sequelae. At a median follow-up of 62 months, side effects related to radiotherapy were rare (3, 27%) and mild (no grade > 2). None of the seven deaths were MCC-related. Our results confirm that radiotherapy can be the curative treatment for eyelid MCC in elderly patients who are not eligible for surgery, or when surgery would be overaggressive because the tumor is already too extended.

In 1999, Kivelä et al. [30] were the first authors to suggest that radiation may be used as the primary and exclusive treatment for eyelid-localized MCC if the patient did not tolerate surgery or the tumor encroached vital structures such as the eye. Overall, only six cases of eyelid lesions were treated with curative radiotherapy (Table 3)**.** Our data compare favorably with those already reported in the literature [28,29,30,31,32].

To our knowledge, we report the largest case series of curative radiotherapy as a treatment strategy for eyelid-localized MCC ≤ 20 mm (T2N0M0), on in-place lesion or after surgery with insufficient oncological margins. Of the 11 patients treated with curative radiotherapy, none have relapsed after this care, and all patients died from unrelated causes. Radiotherapy was well tolerated even in the seven patients treated on the eyelid. On these patients, curative RT treatment without surgery with 5 mm margins led to a control of the pathology.

In the largest case series published to date, Herbert et al., reported the outcomes of 21 patients with MCC of the eyelid [18]. It is the largest cohort evaluating ophthalmologic localisazation of MCC treated with surgery. Some cases were treated with an association of radiation therapy. Similarly to our patients, the majority of their cases with MCC of the eyelid presented with localized eyelid disease. All patients underwent surgery with mostly a wide local excision with margin control (85%) and more than half (57%) received adjuvant radiotherapy for the eyelids, regional nodes, and intervening tissue following a wide local excision. Overall, 6 of the 12 MCC patients (T2aN0M0; 57%), comparable to our cohort, were treated with adjunctive local radiotherapy. One patient developed both regional nodal and distant metastatic recurrent disease 6 months after surgery, even though the primary lesion was small (8mm). This patient did not receive adjunctive radiotherapy. This emphasizes the importance of radiotherapy in the management of MCC. Within a median of 36 months, no other patient with a MCC < 20 mm experienced recurrence. None of their patients were treated with curative radiotherapy [18]. 

Nevertheless, our population is comparable to other published cases of eyelid MCC, particularly with localized lesions < 20 mm as noted by Herbert et al. Our patients treated with curative radiotherapy having similar characteristics to patients of this cohort who received wide local excision with a 5 mm margin and adjunctive radiotherapy did not seem to have a worse prognosis or side effects. The monocentric character, although usually a limitation of the case series, is a guarantee of homogeneity of management in our cohort. One may wonder why none of our patients had SLBN. This procedure was not carried out due to the old age and poor general estate to our patient in balance with the invasive nature of the surgery with a high rate of false negatives in the cervical region.

In our eyelid MCC case series, we found that lesions arise on the upper eyelid rather than the lower. This particularity was also noted in other reviews [14,18]. Authors explain this difference by a higher sun exposure on the upper eyelid [16]. However, basal cell carnicomas are also UV-related lesions, and appear to be more frequent in the lower eyelid [36]. Moreover, we noted that eyebrow MCCs appear to be more frequent in the hair follicle. The origins of MCC remain debatable. Some authors found the origins to be in Merkel cells. These cells are specialized in the light-touch responses derived from epidermal stem cells [37,38] or neural progenitors [39]. In both cases, as follicles are associated with epidermal stem cells and touch receptors, this could explain the localization close to hair follicles on eyebrows or eyelashes on upper eyelid.

In locally advanced MCC, neo-adjuvant chemotherapy had a good outcome [40]. Several cases of spontaneous or post-biopsy regression have been reported, suggesting the possible effectiveness of immunotherapy as in the treatment of melanoma [16,18,41,42]. Checkpoint inhibitors are validated in metastatic MCCs [43,44]. Lipson et al. showed that an endogenous immune response promotes PD-L1 expression in the MCC microenvironment and provides a rationale for investigating therapies blocking PD-1/PD-L1 for patients with MCC, and could be related to overall survival [45].

Guidelines from the NCCN currently recommend consideration of immunotherapy for patients with metastatic MCC or recurrent locally advanced MCC unamenable to definitive resection or RT. Neoadjuvant strategy off protocol is currently not a standard of care [21]. Topalian et al. showed that preoperative anti PD1 for patients with MCPyV-positive, resectable MCC was generally tolerable and induced pathological complete response and radiographic tumor regressions in approximately one half of treated patients [46]. In complex and non-operable MCC of the eyelid in elderly patients with contraindications for receiving chemotherapy, neoadjuvant immunotherapy combined with RT could be interesting.

## 5. Conclusions

To conclude, our results suggest that curative radiotherapy is a safe and effective alternative to surgery for eyelid-localized MCC, in patients with comorbidities or when surgery would lead to aesthetic or functional sequelae. If treated via surgery, rapid local and nodal adjuvant radiotherapy should be considered.

## Figures and Tables

**Table 1:** Patient characteristics.

Variable	n = 11	Percentage %
Median Age (years) (min–max)	77 (53–94)	-
Sex	-	-
Male	4	36
Female	7	64
Associated hemopathy	1	9
Median Size (mm) (min–max)	13.2 (5–20)	-
T1 (≤ 10 mm)	5	45
T2 (≤ 20 mm)	6	54
Localization	-	-
Eyebrow	5	45
Eyelid	6	54
Upper eyelid	5	83
Lower eyelid	1	17

**Table 2:** Treatment and outcomes.

Curative Radiotherapy	n = 11	Percentage %
Surgical biopsy with excision with insufficient margins of 0.1 cm	3	21
Median Lesion dose (Gy) (min–max)	57 (47–70)	-
Lesion boost (nbr)	5	45
Median dose of boost (Gy) (min–max)	12 (8–20)	
Nodal irradiation	9	82
Median nodal dose (Gy) (min–max)	50 (43–51)	-
Hypofracting	3	21
Number of fraction (nbr) (min–max)	15 (10–18)	-
Dose per fraction (Gy) (min–max)	3 (3–6)	-
Median follow up (months) (min–max)	62 (6–152)	-
Side effects	3	27
Local relapse	0	-
Death	7	64
Linked to MCC	0	-

**Table 3:** Review of the literature of eyelid MCC treated with curative radiotherapy.

Author	Lesion	Dose	Follow up
Ashby et al., (1989) [33]	1 case of lower eyelid 1 cm (T1N0M0)	39 Gy (6 × 6.5 Gy)	3 years
Dini et al., (1997) [34]	-	-	2 months
Ott et al., (1999) [31]	1 case of eyebrow 1.7 cm (T2N0M0)	45 Gy	33 months
Ott et al., (1999) [31]	1 case of eyelid 1.2 cm (T2N0M0)	39 Gy	60 months
Sinclair et al., (2003) [32]	1 case of upper eyelid 2 cm (T2N0M0)	40 Gy (15 × 2.6 Gy)	-
Tuskada et al., (2013) [35]	1 case Lower eyelid 5.5 cm (T3N0M0)	50 Gy (5 × 10 Gy) *	6 months
Boileau et al., (2023)	11 cases: - 6 eyelids - 5 eyebrows	57 Gy (range: 47–70)	62 months

* associated with adjuvant nodal conventional irradiation 54 (24 × 2.25 Gy).

## Data Availability

Data are available upon reasonable request to the authors.
